# Taxon-related variation in palmar and plantar friction ridge patterns among *Aotus infulatus*, *Saimiri collinsi* and *Sapajus* spp.: Individual-level variation and implications for identification

**DOI:** 10.1371/journal.pone.0360621

**Published:** 2026-11-11

**Authors:** Juliana Vieira Flores Sales, Rodrigo M. de Barros, Paulo H. G. de Castro, Antonizete R. Souza, Líria Q. L. Hirano

**Affiliations:** 1 Universidade de Brasília (UnB), Brasília, Brazil; 2 Centro de Primatologia do Instituto de Ciências Biológicas UnB, Brazil; 3 Instituto de Identificação do Departamento de Polícia Técnica da Polícia Civil do Distrito Federal (PCDF), Brazil; 4 Centro Nacional de Primatas (CENP), Belém, Brazil; 5 Professor of the Graduate Program in Animal Sciences (PPGCA) UnB, Brazil; Ceuma University: Universidade Ceuma, BRAZIL

## Abstract

Friction ridges play important biomechanical roles in primates, contributing to the regulation of skin moisture and increasing friction with substrates and objects during locomotion and manipulation. In this context, the present study aimed to characterize and compare level I and II dermatoglyphic patterns of the palmar and plantar surfaces of *Aotus infulatus* (owl monkey), *Saimiri collinsi* (squirrel monkey), and *Sapajus* spp. (capuchin monkey). Following chemical restraint, skin impressions of the hands and feet were obtained by applying a thin layer of commercial ink used for human fingerprinting. The analyses revealed a consistent pattern of differentiation among taxa. *A. infulatus* exhibited greater dermatoglyphic homogeneity, with a higher frequency of deltas and whorls, as well as a greater occurrence of the dot on the ridge (P-IN) minutia compared to the other groups. Additionally, no identical palmar or plantar epidermal impressions were observed among individuals within the same taxon, indicating individual-level variability in these structures. Thus, palmar and plantar friction ridge patterns may provide useful morphological markers for comparative studies of taxon-related variation in non-human primates, with potential applications in biological research, population management, and conservation strategies.

## 1. Introduction

New World primates inhabit tropical and subtropical forests of Central and South America and exhibit wide variation in body size, diet, and social organization. Among Neotropical primates, the genera *Aotus* (owl monkeys), *Sapajus* (capuchin monkeys), and *Saimiri* (squirrel monkeys) display distinct activity patterns, dietary strategies, and manipulative abilities. *Aotus* species are nocturnal and exhibit adaptations to low-light environments, including relatively enlarged eyeballs, wide pupils, and retinas with a higher proportion of rod photoreceptors [1,2]. Species of the genus *Saimiri* are diurnal and highly social, presenting remarkable agility for locomotion on slender branches and prey capture [3,4]. In contrast, the genus *Sapajus* is characterized by high encephalization, advanced exploratory manipulation, and tool use in different ecological contexts [5,6].

Friction ridge analysis is based on the interpretation of three levels of detail. Level I corresponds to the analysis of global ridge patterns, also known as dermatoglyphics [7]. Level II refers to minutiae, or Galton details, whose number, position, and orientation are highly informative in friction ridge comparison and have been widely used in human identification contexts [8–10]. Finally, Level III comprises microdetails, particularly sweat pores, fine ridge contours, and ridge edge morphology, which are recognized as highly discriminative features in latent fingerprints and high-resolution systems [11–13].

In non-human primates (NHPs), recent studies involving the black-tufted marmoset (*Callithrix penicillata*) demonstrated that friction ridges present on palmar and plantar surfaces form distinct patterns among individuals. This finding suggests that palmar and plantar friction ridge patterns may retain individual-level variation in non-human primates and may be useful for future biometric applications in scientific research, management programs, and conservation initiatives [14].

The present study aimed to characterize and compare Level I and Level II dermatoglyphic patterns [15] of the palmar and plantar surfaces of *A. infulatus* (Kuhl, 1820), *S. collinsi* (Osgood, 1916), and *Sapajus* spp. (Kerr, 1792). The hypotheses tested were I, dermatoglyphics (Level I), deltas, and minutiae (Level II) exhibit distinct patterns among taxa; II, palmar and plantar dermatoglyphic patterns exhibit non-coincident configurations among individuals within the analyzed sample, indicating individual-level variability.

## 2. Materials and methods

### 2.1. Animals and authorizations

Non-human primates of the species *A. infulatus*, *S. collinsi*, and *Sapajus* spp., including juveniles and adults of both sexes maintained under human care, were evaluated in this study (Table 1).

**Table 1:** Characterization of the non-human primates included in the study according to taxonomy, origin, sex, and life stage.

Taxon	Sex	Life stage^1^	Total
*Aotus infulatus* (owl monkey)	F: 16 (45.7%) M:19 (54.3%)	J: 3 (8.6%); SubA: 4 (11.4%) Ad: 28 (80%)	35 (100%)
*Saimiri collinsi* (squirrel monkey)	F: 14 (40%) M: 21 (60%)	SubA: 4 (11.4%) Ad: 31 (88.6%)	35 (100%)
*Sapajus* spp. (capuchin monkey)	F: 23 (50%) M: 23 (50%)	Ad: 46 (100%)	46 (100%)

Legend: Ad: adult; F: female; J: juvenile; M: male; SubA: subadult. ^1^Source: Auricchio [1].

All procedures were conducted in accordance with current ethical approvals and guidelines (IEC: 17/2024; CEUA/UnB: SEI 23106.126774/2023–81). Regarding *A. infulatus* and *S. collinsi*, the study was conducted at the National Primate Center, Ananindeua, Pará, Brazil. Samples of *Sapajus* spp. were collected from animals belonging to the UnB Primate Center and from individuals admitted to the Wildlife Sector of the Veterinary Hospital of the University of Brasília (HVet-UnB), Brasília, Federal District, Brazil.

At the UnB Primate Center and the National Primate Center, animals were housed in spacious enclosures located adjacent to vegetated areas, either in pairs or in small social groups, under natural photoperiod, temperature, and humidity conditions. The enclosures included designated resting areas and provided physical, dietary, and social enrichment. Animals were fed a commercial primate diet supplemented with fresh fruits and vegetables, with water available ad libitum. At HVet-UnB, primates were housed in enclosures appropriate for their body size and clinical condition and were provided with a nutritionally appropriate diet, water ad libitum, and environmental enrichment.

Epidermal print collection is noninvasive and does not involve surgical or painful procedures. Samples were obtained only during previously scheduled clinical or management procedures that already require chemical restraint, thereby avoiding additional anesthesia solely for research purposes. Animals were fasted for six hours before chemical restraint and were continuously monitored by the veterinary team throughout the procedure. Sample collection was immediately discontinued if any complication or indication of compromised animal welfare is observed. When clinically indicated, supportive measures included discontinuation of inhalant anesthesia, supplemental oxygen, and active thermal support. Following sample collection, animals were monitored for three days for potential hypersensitivity or other adverse reactions to the fingerprint ink.

No animals were euthanized for the purposes of this study. Animals housed at the primate centers remain at their respective institutions after sample collection, whereas animals treated at HVet-UnB were released, transferred, or otherwise assigned an appropriate legal destination after veterinary discharge, in accordance with applicable Brazilian legislation.

### 2.2. Epidermal print collection

Sample collection was performed during routine management procedures conducted by the institutions to minimize stress and reduce the risks associated with chemical restraint. For epidermal print acquisition, animals were chemically restrained according to the anesthetic protocols established by each technical team.

For *A. infulatus* and *S. collinsi*, tiletamine and zolazepam (5 mg/kg) were administered intramuscularly (IM). In *Sapajus* spp., either inhalation anesthesia with isoflurane administered via facial mask using a portable anesthetic device with a universal vaporizer, or ketamine (7 mg/kg IM) combined with midazolam (0.3 mg/kg IM), was used.

Epidermal prints were collected after confirmation of muscle relaxation. Palmar and plantar surfaces were cleaned with water and dried with paper towels to remove debris. Subsequently, a thin layer of commercial ink designed for human fingerprint collection (Fingerprint Ink Sirchie^®^ Cat. No. 231T, Sirchie Fingerprint Laboratories, Youngsville, NC, USA) was applied using a 5-cm foam roller. Excess ink was removed with paper towels, and the palmar or plantar surface of interest was then gently pressed onto a white A4 sheet of paper.

For analysis, the sheets containing the friction ridge impressions were scanned at a resolution of 2400 dpi. After digitization, the images were evaluated for the macroscopic description of ridge patterns (Level I) and minutiae (Level II) [9,14–16].

To standardize anatomical descriptions and image analysis, palmar and plantar impressions were oriented with the digits facing upward and the proximal portion of the limb facing downward. Medial and lateral directions were defined according to the anatomical orientation of each limb, and right and left sides corresponded to the respective antimer of the animal.

### 2.3. Level I dermatoglyphic analysis

Level I dermatoglyphic analysis was performed in three regions of the palmar impressions (interdigital, thenar, and hypothenar) and in four regions of the plantar impressions (interdigital, thenar, hypothenar, and calcaneal). Friction ridge patterns observed in the palmar and plantar impressions were classified as arches, loops, and whorls.

In the arch pattern, friction ridges exhibit a simple arrangement and traverse the area from one side to the other. In the loop pattern, ridges form an open circuit, emerging from one side, curving, and returning toward a point near their origin. Finally, the whorl pattern is characterized by the formation of a closed or nearly closed circuit, in which the ridges form concentric, or spiral turns around a central core. In addition to quantifying arches, loops, and whorls, the number of deltas in the described regions was also recorded [15,17–19] (Fig 1).

**Fig 1:**
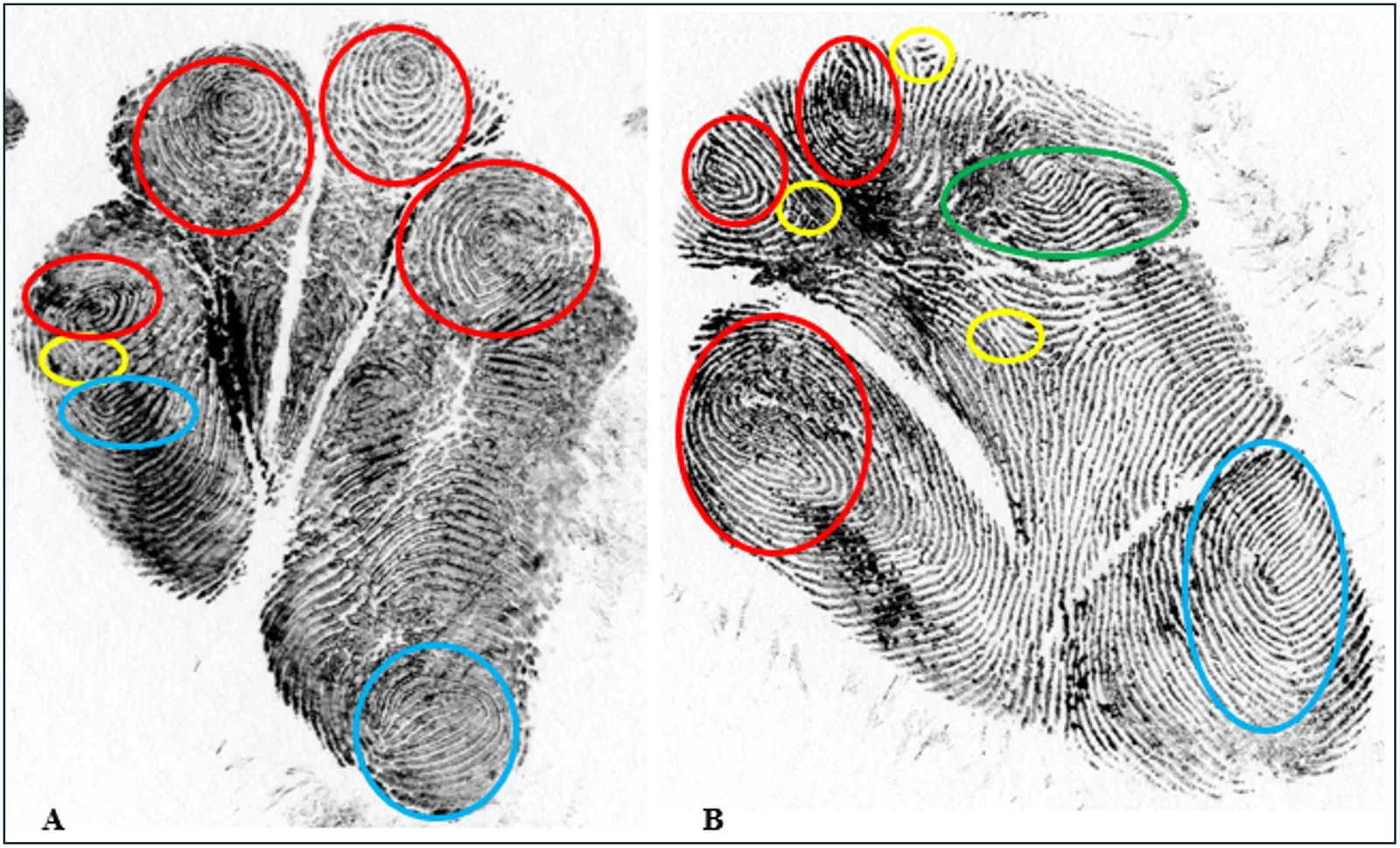
Level I dermatoglyphic analysis of the right palms of *Aotus infulatus* (A) and *Sapajus* spp. (B). Legend: red, whorl; green, arch; blue, loop; yellow, delta.

### 2.4. Level II dermatoglyphic analysis

Level II dermatoglyphic analysis of the friction ridge impressions was performed exclusively in the hypothenar region defined as the palmar area adjacent to the ulna, extending from the base of the wrist distally toward the fifth digit. In the plantar surface, this area corresponds to the lateral portion extending proximally from the fifth digit to the calcaneal region [17,20]. The hypothenar region was selected because it presented the best sample quality, and the analysis followed the sequence of the left distal, right distal, and proximal areas.

Among the images obtained, Level II dermatoglyphic analysis was successfully performed on 68 forelimb/thoracic limb (TL) impressions and 66 hindlimb/pelvic limb (PL) impressions for *A. infulatus*, 25 TL and 21 PL impressions for *S. collinsi*, and 54 TL and 40 PL impressions for *Sapajus* spp. Some samples were excluded due to insufficient image sharpness, generally caused by excess ink or smearing artifacts.

Minutiae analysis and counting in the palmar and plantar scans were performed using a web application developed in HTML, CSS, and JavaScript and executed in Google Chrome browser. The designation “OBS” was used to record rare minutiae not included in the software database, and these were identified separately in Microsoft Excel 365^®^ spreadsheets. Minutiae markings were sequentially numbered within each class.

### 2.5. Assessment of individual-level dermatoglyphic variation

To assess individual-level variation, 12 minutiae per epidermal impression were counted and mapped as an operational criterion for comparing configurations among individuals. This threshold was adopted based on quantitative practices historically used in human friction ridge comparison, including in Brazil, and has also been applied in previous studies involving non-human primates [14,21].

### 2.6. Statistical analysis

In Level I dermatoglyphic evaluations, the anatomical impression was used as the sampling unit, and differences among taxa were analyzed using PERMANOVA based on Jaccard index. PERMANOVA and BETADISPER were performed using the same set of available impressions for multivariate analysis. In Level II dermatoglyphic evaluations, differences in minutiae composition were analyzed using PERMANOVA based on Bray-Curtis dissimilarity. For comparisons involving taxon, sex, and age category, minutiae count from the available anatomical impressions were aggregated at the individual level, and the individual animal was used as the sampling unit. Anatomical effects, including region (palmar or plantar), antimer (left or right), and their interaction, were evaluated using the available impressions, with permutations restricted within individuals to account for repeated measurements. The analytical N, expressed as the number of animals or anatomical impressions according to the sampling unit used, is reported with the corresponding results. The individual-level data underlying the descriptive and statistical analyses are provided in S2 File.

The influence of extreme values in Level II data was investigated using Hellinger transformation followed by PERMANOVA based on Euclidean distances. As no qualitative differences were observed between the results, analyses based on the original matrix were retained. For both levels, homogeneity of multivariate dispersions among taxa was assessed using BETADISPER, based on the distances of individual observations to the centroid of their respective taxon. Differences in dispersion were evaluated using the global BETADISPER test, followed by Tukey’s post-hoc pairwise comparisons when significant. Multivariate patterns were visually inspected using non-metric multidimensional scaling (nMDS). Finally, the contribution of variables to dissimilarity among taxa was estimated using similarity percentage analysis (SIMPER).

All statistical analyses were performed in R statistical software [22] using the packages vegan, rstatix, corrplot, and ggplot2. A significance level of p < 0.05 was adopted.

## 3. Results

### 3.1. Level I dermatoglyphic analysis

#### 3.1.1. Palmar impressions.

The results of the Level I dermatoglyphic analysis are presented in Table 2. Regarding the frequency of dermatoglyphic patterns in palmar impressions, based on the mean values obtained from the three regions analyzed (interdigital, thenar, and hypothenar), the whorl pattern predominated in all three taxa, with frequencies of 99.5% in *A. infulatus*, 80% in *S. collinsi*, and 60.5% in *Sapajus* spp. Regarding deltas, frequencies of 99.5% were observed in *A. infulatus*, 84.8% in *S. collinsi*, and 82.6% in *Sapajus* spp.

**Table 2:** Frequencies of dermatoglyphics and deltas in palmar epidermal impressions of *Aotus infulatus*, *Saimiri collinsi*, and *Sapajus* spp.

R	D	LTL	RTL	TL
*Aotus infulatus*				
**Interdigital**	Ar	5/35 (14.3%); 0.14 ± 0.35	4/35 (11.4%); 0.11 ± 0.32	9/70 (12.9%); 0.13 ± 0.34^*^
	Lo	0/35 (0)	1/35 (2.9%); 0.03 ± 0.17	1/70 (1.4%); 0.01 ± 0.12^*+^
	Wh	35/35 (100%); 3.09 ± 0.28	35/35 (100%); 3.09 ± 0.28	70/70 (100%); 3.09 ± 0.28
	Dl	35/35 (100%); 4.6 ± 0.98	35/35 (100%); 4.94 ± 1.03	70/70 (100%); 4.77 ± 1.01^+^
**Thenar**	Ar	1/35 (2.9%); 0.03 ± 0.17	1/35 (2.9%); 0.03 ± 0.17	2/70 (2.9%); 0.03 ± 0.17
	Lo	13/35 (37.1%); 0.37 ± 0.49	16/35 (45.7%); 0.46 ± 0.50	29/70 (41.4%); 0.41 ± 0.5^*+^
	Wh	35/35 (100%); 1.37 ± 0.49	35/35 (100%); 1.23 ± 0.43	70/70 (100%); 1.3 ± 0.46^*^
	Dl	35/35 (100%); 2.49 ± 0.82	34/35 (97.1%); 2.06 ± 0.80	69/70 (98.6%); 2.27 ± 0.83^+^
**Hypothenar**	Ar	1/35 (2.9%); 0.03 ± 0.17	2/35 (5.7%); 0.06 ± 0.23	3/70 (4.3%); 0.04 ± 0.2
	Lo	35/35 (100%); 1 ± 0	35/35 (100%); 1 ± 0	70/70 (100%); 1 ± 0
	Wh	35/35 (100%); 2.83 ± 0.45	34/35 (97.1%); 2.71 ± 0.67	69/70 (98.6%); 2.77 ± 0.57^*+^
	Dl	35/35 (100%); 5.2 ± 1.21	35/35 (100%); 4.91 ± 1.60	70/70 (100%); 5.06 ± 1.41^*+^
*Saimiri collinsi*				
**Interdigital**	Ar	0/35 (0)	0/35 (0)	0/70 (0)
	Lo	0/35 (0)	0/35 (0)	0/70 (0) ^+^
	Wh	32/35 (91.4%); 2.63 ± 0.88	35/35 (100%); 2.91 ± 0.37	67/70 (95.7%); 2.77 ± 0.68^#^
	Dl	26/35 (74.3%); 2.29 ± 1.53	27/35 (77.1%); 2.49 ± 1.65	53/70 (75.7%); 2.39 ± 1.58 ^+ #^
**Thenar**	Ar	0/35 (0)	0/35 (0)	0/70 (0)
	Lo	0/35 (0)	1/35 (2.9%); 0.03 ± 0.17	1/70 (1.4%); 0.01 ± 0.12^+^
	Wh	31/35 (88.6%); 0.89 ± 0.32	34/35 (97.1%); 1 ± 0.24	65/70 (92.3%); 0.94 ± 0.29
	Dl	31/35 (88.6%); 1.23 ± 0.69	31/35 (88.6%); 1.31 ± 0.72	62/70 (88.6%); 1.27 ± 0.7 ^+ #^
**Hypothenar**	Ar	2/35 (5.7%); 0.06 ± 0.23	0/35 (0)	2/70 (2.9%); 0.03 ± 0.17
	Lo	29/35 (82.9%); 0.83 ± 0.38	27/35 (77.1%); 0.77 ± 0.43	56/70 (80%); 0.8 ± 0.4^#^
	Wh	22/35 (62.9%); 0.71 ± 0.99	14/35 (40%); 0.69 ± 0.96	36/70 (51.4%); 0.7 ± 0.97^+^
	Dl	32/35 (91.4%); 1.74 ± 1.04	31/35 (88.6%); 1.66 ± 1.14	63/70 (90%); 1.7 ± 1.08^+^
*Sapajus* spp.				
**Interdigital**	Ar	2/46 (4.3%); 0.04 ± 0.2	1/46 (2.17%); 0.02 ± 0.15	3/92 (3.3%); 0.03 ± 0.18^*^
	Lo	0/46 (0)	0/46 (0)	0/92 (0)^*^
	Wh	40/46 (86.7%); 2.48 ± 1.05	39/46 (84.8%); 2.48 ± 1.09	79/92 (85.9%); 2.48 ± 1.06^#^
	Dl	39/46 (84.8%); 3.30 ± 1.93	38/46 (82.6%); 3.39 ± 1.9	77/92 (83.4%); 3.35 ± 1.9^#^
**Thenar**	Ar	0/46 (0)	2/46 (4.3%); 0.09 ± 0.41	2/92 (2.17%); 0.04 ± 0.29
	Lo	7/46 (15.2%); 0.15 ± 0.36	6/46 (13.0%); 0.15 ± 0.42	13/92 (14.1%); 0.15 ± 0.39^*^
	Wh	30/46 (65.2%); 0.67 ± 0.52	27/46 (58.7%); 0.61 ± 0.54	57/92 (62%); 0.64 ± 0.53^*^
	Dl	37/46 (80.4%); 1.52 ± 1.11	37/46 (80.4%); 1.30 ± 0.87	74/92 (80.4%); 1.41 ± 1.0^#^
**Hypothenar**	Ar	0/46 (0)	1/46 (2.17%); 0.02 ± 0.15	1/92 (1.1%); 0.01 ± 0.1
	Lo	23/46 (50%); 0.65 ± 0.85	22/46 (47.8%); 0.52 ± 0.59	45/92 (48.9%); 0.59 ± 0.73^#^
	Wh	16/46 (34.8%); 0.39 ± 0.61	15/46 (32.6%); 0.37 ± 0.61	31/92 (33.7%); 0.38 ± 0.61^*^
	Dl	38/46 (82.6%); 1.72 ± 1.22	39/46 (84.8%); 2.06 ± 1.24	77/92 (83.4%); 1.89 ± 1.23^*^

Legend: *Indicates a statistically significant difference (p < 0.05) between *A. infulatus* and *Sapajus* spp.; + Indicates a statistically significant difference (p < 0.05) between *A. infulatus* and *S. collinsi*; #Indicates a statistically significant difference (p < 0.05) between *Sapajus* spp. and *S. collinsi*; Ar: arch; D: dermatoglyphic; Dl: delta; Lo: loop; LTL: left thoracic limb; TL: thoracic limbs (right and left); R: region; RTL: right thoracic limb; Wh: whorl. Data are presented as: frequency of animals exhibiting the dermatoglyphic/total number of samples (%); mean ± standard deviation of the number of dermatoglyphics and deltas.

Level I results for palmar impressions showed statistically significant differences according to species (F_2,212_ = 48.391; p = 0.001) and age category (F_2,212_ = 6.668; p = 0.001). However, the low coefficient of determination (R^2^) for age category (R^2^ = 0.02) indicated limited explanatory power. Therefore, the predictive effect of this variable was excluded, and only species (R^2^ = 0.30) was considered in the evaluation of the data.

The variable that best discriminated *A. infulatus* from *Sapajus* spp. and *S. collinsi* in palmar impressions was the number of deltas in the hypothenar region, with SIMPER-estimated mean dissimilarities of 0.09 ± 0.05 (p = 0.001) and 0.11 ± 0.05 (p = 0.001), respectively. Between *Sapajus* spp. and *S. collinsi*, the most relevant variable was the number of deltas in the interdigital region, with a mean dissimilarity of 0.09 ± 0.08 (p = 0.001) (Table 3, S1 Table in S1 File).

**Table 3:** Variables with significant contribution (p < 0.05) to the dissimilarity among *Aotus infulatus*, *Saimiri collinsi*, and *Sapajus* spp. in the Level I palmar dermatoglyphic analysis.

Taxa	Region/ Level I analysis	Mean	SD	Ratio	p*
*Aotus × Sapajus*	Hypothenar/delta	0.0896	0.0491	1.82	<0.001
	Hypothenar/whorl	0.0706	0.0239	2.96	<0.001
	Thenar/whorl	0.0175	0.0188	0.93	0.001
	Thenar/loop	0.0137	0.0156	0.88	<0.001
	Interdigital/arch	0.0048	0.0112	0.43	0.002
	Interdigital/loop	0.0004	0.0034	0.12	<0.001
*Aotus × Saimiri*	Hypothenar/delta	0.1090	0.0527	2.06	<0.001
	Interdigital/delta	0.0819	0.0609	1.35	<0.001
	Hypothenar/whorl	0.0690	0.0358	1.93	<0.001
	Thenar/delta	0.0379	0.0291	1.30	0.018
	Thenar/loop	0.0134	0.0161	0.83	<0.001
	Interdigital/loop	0.0004	0.0036	0.12	<0.001
*Sapajus × Saimiri*	Interdigital/delta	0.0920	0.0782	1.18	<0.001
	Thenar/delta	0.0379	0.0363	1.04	0.010
	Hypothenar/loop	0.0250	0.0269	0.93	<0.001
	Interdigital/whorl	0.0183	0.0397	0.46	<0.001

Legend: *p-values were obtained from permutation tests associated with the SIMPER analysis. Mean represents the average contribution of each variable to the dissimilarity between groups; SD is the standard deviation of this contribution; Ratio = Mean/SD. Only variables with p < 0.05 are shown.

Multivariate dispersion differed significantly among taxa (BETADISPER: F_2_,_108_ = 17.837; p < 0.001; N = 111 palmar impressions). *A. infulatus* presented the lowest mean distance to centroid (0.143), whereas *S. collinsi* and *Sapajus* spp. showed values of 0.243 and 0.252, respectively. Pairwise Tukey comparisons indicated significantly lower dispersion in *Aotus* than in *S. collinsi* (mean difference = 0.100; p < 0.001) and *Sapajus* spp. (mean difference = 0.104; p < 0.001), whereas *S. collinsi* and *Sapajus* spp. did not differ significantly (p = 0.991). In addition to exhibiting lower intrataxon variation, *A. infulatus* also differed morphologically from the other taxa, which appeared more like each other (Fig 2).

**Fig 2:**
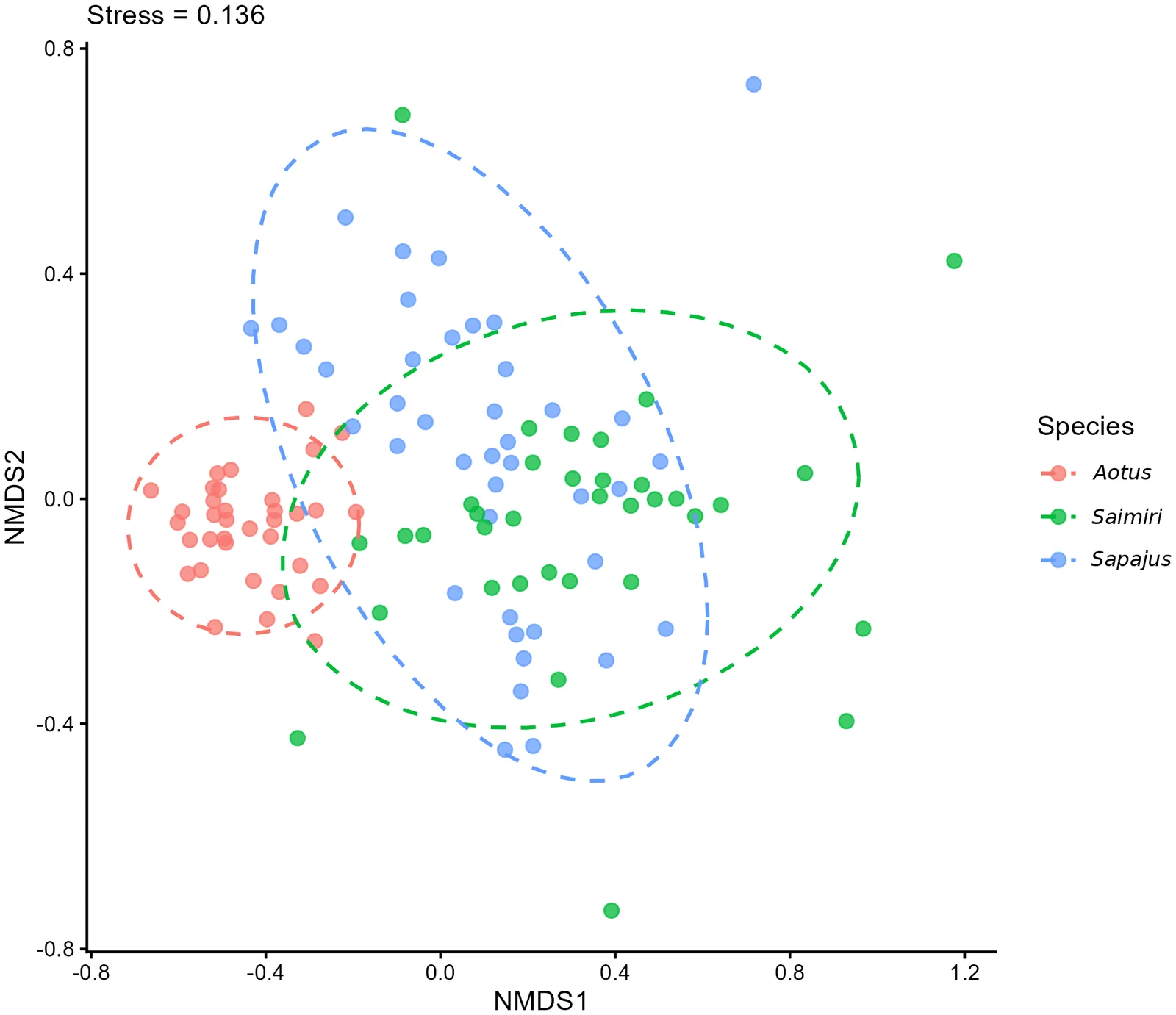
Distances derived from dermatoglyphic and delta analyses of the palmar limbs of *Aotus infulatus*, *Saimiri collinsi*, and *Sapajus* spp. Each point represents one individual. Points with different colors and shapes represent samples from different taxa and antimeres, respectively. Stress indicates the goodness-of-fit of the non-metric multidimensional scaling (nMDS) model. Source: Graph produced using R Core Team software [22].

#### 3.1.2. Plantar impressions.

The left pelvic limb plantar impression of one male *S. collinsi* specimen was excluded from the analysis due to insufficient image sharpness. Four plantar regions (interdigital, thenar, hypothenar, and calcaneal) were evaluated in the three taxa. However, the calcaneal region of all *A. infulatus* specimens exhibited a hyperkeratinized structure resembling a callus, which prevented the analysis of this region in the species (Fig 3). The institution’s technical staff associated this alteration with the prolonged time spent by the animals in enclosure nests with mesh flooring and on wooden platforms within the enclosures, since this condition is not observed in free-ranging individuals.

**Fig 3:**
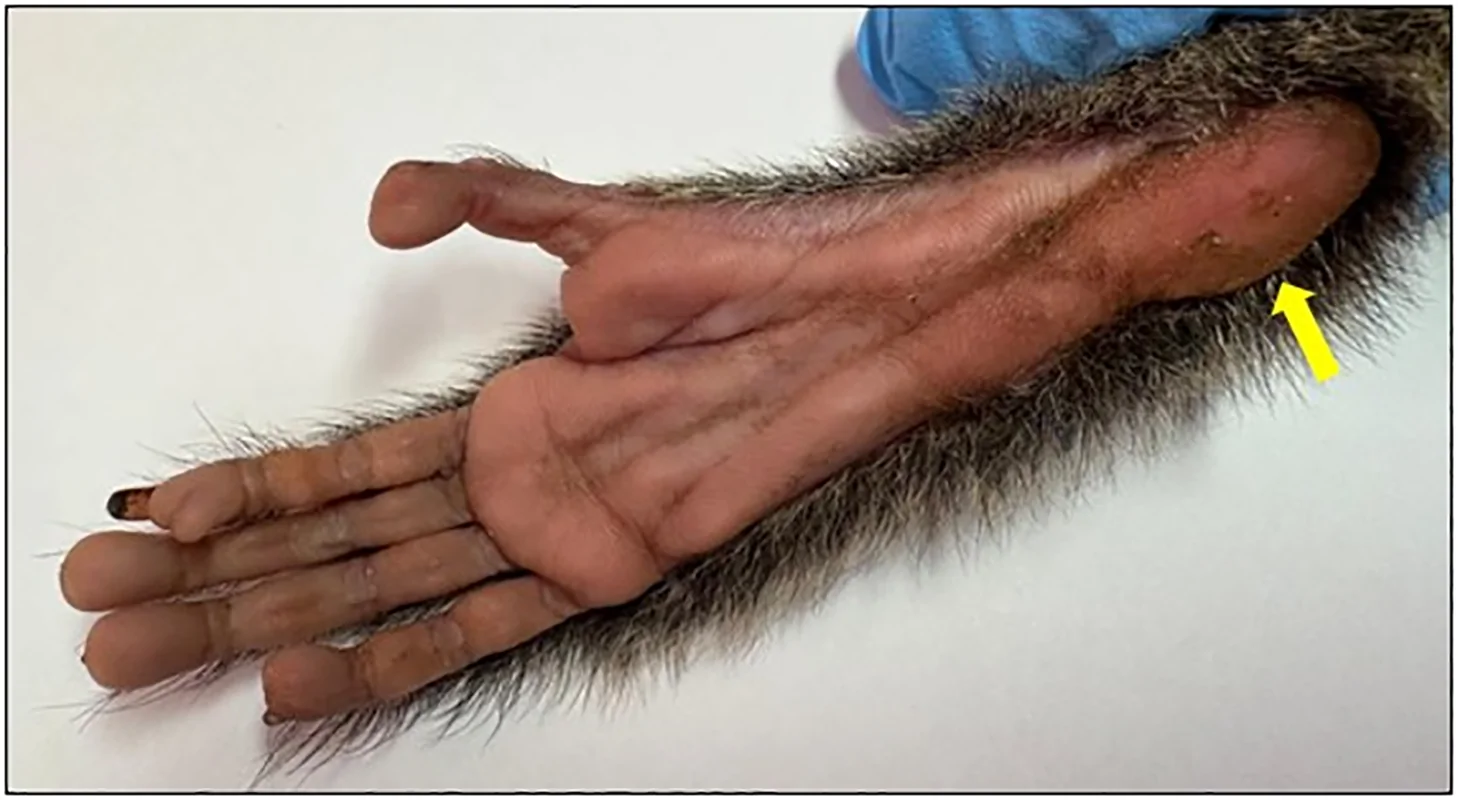
Macrophotograph of the right plantar surface of *Aotus infulatus.* Note the hyperkeratinization in the calcaneal region (arrow).

The dermatoglyphic patterns and deltas observed in the plantar epidermal impressions are presented in Table 4. Regarding the frequency of dermatoglyphic patterns and deltas in the plantar impressions, based on the mean values obtained from the four regions analyzed together, the whorl pattern predominated in *A. infulatus* (96.7%). In contrast, loops were more frequently observed in *Sapajus* spp. (41.03%) and *S. collinsi* (46.4%). Regarding deltas, frequencies of 100% were observed in *A. infulatus*, 67% in *S. collinsi*, and 59% in *Sapajus* spp.

**Table 4:** Frequencies of dermatoglyphics in plantar epidermal impressions of Aotus infulatus, Saimiri collinsi, and Sapajus spp.

R	D	LPL	RPL	PL
*Aotus infulatus*				
Interdigital	Ar	0/35 (0)	0/35 (0)	0/70
	Lo	34/35 (97.1%); 1 ± 0.24	29/35 (82.9%); 0.83 ± 0.38	63/70 (90%); 0.91 ± 0.33
	Wh	35/35 (100%); 2.6 ± 0.5	35/35 (100%); 2.66 ± 0.54	70/70 (100%); 2.63 ± 0.52^+^
	Dl	35/35 (100%); 4.8 ± 1.13	35/35 (100%); 4.66 ± 0.91	70/70 (100%); 4.73 ± 1.02^+^
Thenar	Ar	0/35 (0)	0/35 (0)	0/70 (0)
	Lo	31/35 (88.6%); 1.34 ± 0.76	32/35 (91.4%); 1.37 ± 0.77	63/70 (90%); 1.38 ± 0.76
	Wh	34/35 (97.1%); 1.74 ± 0.82	29/35 (82.9%); 1.51 ± 1.04	63/70 (90%); 1.63 ± 0.93^*+^
	Dl	35/35 (100%); 4.98 ± 1.5	35/35 (100%); 4.57 ± 1.58	70/70 (100%); 4.77 ± 1.54^+^
Hypothenar	Ar	0/35 (0)	0/35 (0)	0/70 (0)
	Lo	32/35 (91.4%); 1.46 ± 0.89	34/35 (97.1%); 1.71 ± 0.82	66/70 (94.3%); 1.59 ± 0.86
	Wh	35/35 (100%); 2.94 ± 1.5	35/35 (100%); 2.69 ± 1.13	70/70 (100%); 2.81 ± 1.33^*+^
	Dl	35/35 (100%); 6.5 ± 2.4	35/35 (100%); 5.77 ± 1.86	70/70 (100%); 6.16 ± 2.19^*+^
Calcaneal	Ar	N/A	N/A	N/A
	Lo	N/A	N/A	N/A
	Wh	N/A	N/A	N/A
	Dl	N/A	N/A	N/A
*Saimiri collinsi*				
Interdigital	Ar	0/34 (0)	0/35 (0)	0/69 (0) ^#^
	Lo	20/34 (58.8%); 0.73 ± 0.71	18/35 (51.4%); 0.71 ± 0.82	38/69 (55.1%); 0.72 ± 0.76^#^
	Wh	15/34 (44.1%); 0.65 ± 0.81	23/35 (65.7%); 1.03 ± 0.86	38/69 (55.1%); 0.84 ± 0.85^+#^
	Dl	26/34 (76.5%); 1.53 ± 1.16	30/35 (85.7%); 1.94 ± 1.11	56/69 (81.2%); 1.74 ± 1.15^+#^
Thenar	Ar	0/34	0/35 (0)	0/69 (0) ^#^
	Lo	27/34 (79.4%); 1.32 ± 0.84	26/35 (74.3%); 1.25 ± 0.85	53/69 (76.8%); 1.29 ± 0.84^#^
	Wh	9/34 (26.5%); 0.29 ± 0.52	10/35 (28.6%); 0.37 ± 0.65	19/69 (27.5%); 0.33 ± 0.59^+^
	Dl	30/34 (88.2%); 1.85 ± 0.99	30/35 (85.7%); 1.97 ± 1.27	60/69 (87%); 1.91 ± 1.13^+#^
Hypothenar	Ar	0/34 (0)	0/35 (0)	0/69 (0) ^#^
	Lo	20/34 (58.8%); 0.56 ± 0.5	17/35 (48.6%); 0.51 ± 0.56	37/69 (53.6%); 0.54 ± 0.53^#^
	Wh	9/34 (26.5%); 0.38 ± 0.7	16/35 (45.7%); 0.71 ± 0.89	25/69 (36.2%); 0.55 ± 0.81^+^
	Dl	28/34 (82.3%); 1.26 ± 0.86	30/35 (85.7%); 1.89 ± 1.34	58/69 (84.1%); 1.58 ± 1.17^+^
Calcaneal	Ar	0/34 (0)	0/35 (0)	0/69 (0)
	Lo	0/34 (0)	0/35 (0)	0/69 (0)
	Wh	0/34 (0)	0/35 (0)	0/69 (0)
	Dl	5/34 (14.7%); 0.21 ± 0.54	6/35 (17.1%); 0.26 ± 0.61	11/69 (15.9%); 0.23 ± 0.57
*Sapajus* spp.				
Interdigital	Ar	4/46 (8.7%); 0.09 ± 0.28	2/46 (4.3%); 0.04 ± 0.21	6/92 (6.5%); 0.06 ± 0.25^#^
	Lo	23/46(50%);0.85±1.03	22/46 (47.8%); 0.61 ± 0.74	45/92 (48.9%); 0.73 ± 0.9^#^
	Wh	18/46 (39.1%); 0.74 ± 0.98	23/46 (50%); 0.93 ± 0.98	41/92 (44.6%); 0.84 ± 0.98^#^
	Dl	33/46 (71.7%); 2.13 ± 1.73	33/46 (71.7%); 2.07 ± 1.66	66/92 (71.7%); 2.1 ± 1.7^#^
Thenar	Ar	4/46 (8.7%); 0.09 ± 0.28	2/46 (4.3%); 0.04 ± 0.21	6/92 (6.5%); 0.06 ± 0.25^#^
	Lo	30/46 (65.2%); 1.37 ± 1.2	26/46 (56.5%); 1.33 ± 1.4	56/92 (60.9%); 1.35 ± 1.3^#^
	Wh	7/46 (15.2%); 0.24 ± 0.6	9/46 (19.6%); 0.35 ± 0.77	16/92 (17.4%); 0.29 ± 0.7^*^
	Dl	35/46 (76.09%); 2.5 ± 1.7	32/46 (69.6%); 2.4 ± 2.0	67/92 (72.8%); 2.44 ± 1.85^#^
Hypothenar	Ar	6/46 (13.04%);0.13 ± 0.34	3/46 (6.5%); 0.11 ± 0.48	9/92 (9.8%); 0.12 ± 0.41^#^
	Lo	25/46 (54.3%); 0.96 ± 1.03	23/46 (50%); 0.74 ± 0.93	48/92 (52.2%); 0.85 ± 0.98^#^
	Wh	4/46 (8.7%);0.15 ± 0.56	6/46 (13%); 0.15 ± 0.42	10/92 (10.9%); 0.15 ± 0.49^*^
	Dl	28/46 (60.9%);1.72 ± 1.68	30/46 (65.2%); 1.63 ± 1.43	58/92 (63%); 1.67 ± 1.55^*^
Calcaneal	Ar	0/46 (0)	0/46 (0)	0/92 (0)
	Lo	1/46 (2.17%);0.02 ± 0.15	1/46 (2.17%); 0.02 ± 0.15	2/92 (2.17%); 0.02 ± 0.15
	Wh	0/46 (0)	3/46 (6.5%); 0.06 ± 0.25	3/92 (3.3%); 0.03 ± 0.18
	Dl	13/46 (28.3%); 0.35 ± 0.6	13/46 (28.3%); 0.46 ± 0.86	26/92 (28.3%); 0.4 ± 0.74

Legend: *Indicates a statistically significant difference (p < 0.05) between *A. infulatus* and *Sapajus* spp.; + Indicates a statistically significant difference (p < 0.05) between *A. infulatus* and *S. collinsi*; # Indicates a statistically significant difference (p < 0.05) between *Sapajus* spp. and *S. collinsi*; Ar: arch; D: dermatoglyphic; Dl: delta; Lo: loop; LPL: left pelvic limb; N/A: not analyzed because the calcaneal region could not be adequately evaluated due to hyperkeratinization; PL: pelvic limbs (right and left); R: region; RPL: right pelvic limb; Wh: whorl. Data are presented as: frequency of animals exhibiting the dermatoglyphic/total number of samples (%); mean ± standard deviation of the number of dermatoglyphics and deltas.

Regarding the comparison of plantar dermatoglyphic patterns and deltas, a statistically significant difference was observed only among taxa (F_2,203_ = 33.549; p = 0.001), with a high coefficient of determination (R^2^ = 0.24). The variable that most strongly contributed to discriminating *A. infulatus* from *Sapajus* spp. and *S. collinsi* was the number of deltas in the hypothenar region, with SIMPER-estimated mean dissimilarities of 0.10 ± 0.06 (p = 0.001) and 0.13 ± 0.06 (p = 0.001), respectively. Between *Sapajus* spp. and *S. collinsi*, the most relevant variable was the number of deltas in the thenar region, with a mean dissimilarity of 0.08 ± 0.07 (p = 0.001) (Table 5, S2 Table in S1 File).

**Table 5:** Variables with significant contribution (p < 0.05) to the dissimilarity among *Aotus infulatus*, *Saimiri collinsi*, and *Sapajus* spp. in the Level I plantar dermatoglyphic analysis.

Taxa	Region/ Level I analysis	Mean	SD	Ratio	p*
*Aotus × Sapajus*	Hypothenar/delta	0.1030	0.0625	1.65	<0.001
	Hypothenar/whorl	0.0655	0.0332	1.97	<0.001
	Thenar/whorl	0.0361	0.0241	1.50	<0.001
*Aotus × Saimiri*	Hypothenar/delta	0.1260	0.0620	2.03	<0.001
	Interdigital/delta	0.0847	0.0471	1.80	<0.001
	Thenar/delta	0.0814	0.0502	1.62	<0.001
	Hypothenar/whorl	0.0650	0.0390	1.67	<0.001
	Interdigital/whorl	0.0509	0.0308	1.65	<0.001
	Thenar/whorl	0.0386	0.0264	1.46	<0.001
*Sapajus × Saimiri*	Thenar/delta	0.0819	0.0656	1.25	<0.001
	Interdigital/delta	0.0743	0.0639	1.16	<0.001
	Thenar/loop	0.0554	0.0473	1.17	<0.001
	Interdigital/whorl	0.0452	0.0437	1.04	0.001
	Interdigital/loop	0.0398	0.0399	1.00	<0.001
	Hypothenar/loop	0.0390	0.0369	1.06	<0.001
	Calcaneal/delta	0.0282	0.0437	0.65	<0.001
	Hypothenar/arch	0.0062	0.0182	0.34	<0.001
	Interdigital/arch	0.0034	0.0118	0.29	<0.001
	Thenar/arch	0.0033	0.0114	0.29	<0.001
	Calcaneal/whorl	0.0019	0.0097	0.19	<0.001
	Calcaneal/loop	0.0010	0.0061	0.17	<0.001

Legend: *p-values were obtained from permutation tests associated with the SIMPER analysis. Mean represents the average contribution of each variable to the dissimilarity between groups; SD is the standard deviation of this contribution; Ratio = Mean/SD. Only variables with p < 0.05 are shown.

Similar to the pattern observed for palmar limbs, multivariate dispersion differed significantly among taxa in plantar limbs (BETADISPER: F_2,104_ = 23.542; p < 0.001; N = 107 plantar impressions). *A. infulatus* presented the lowest mean distance to centroid (0.204), whereas *S. collinsi* and *Sapajus* spp. showed values of 0.374 and 0.378, respectively. Pairwise Tukey comparisons indicated significantly lower dispersion in *A. infulatus* than in *S. collinsi* (mean difference = 0.170; p < 0.001) and *Sapajus* spp. (mean difference = 0.174; p < 0.001), whereas *S. collinsi* and *Sapajus* spp. did not differ significantly (p = 0.991) (Fig 4).

**Fig 4:**
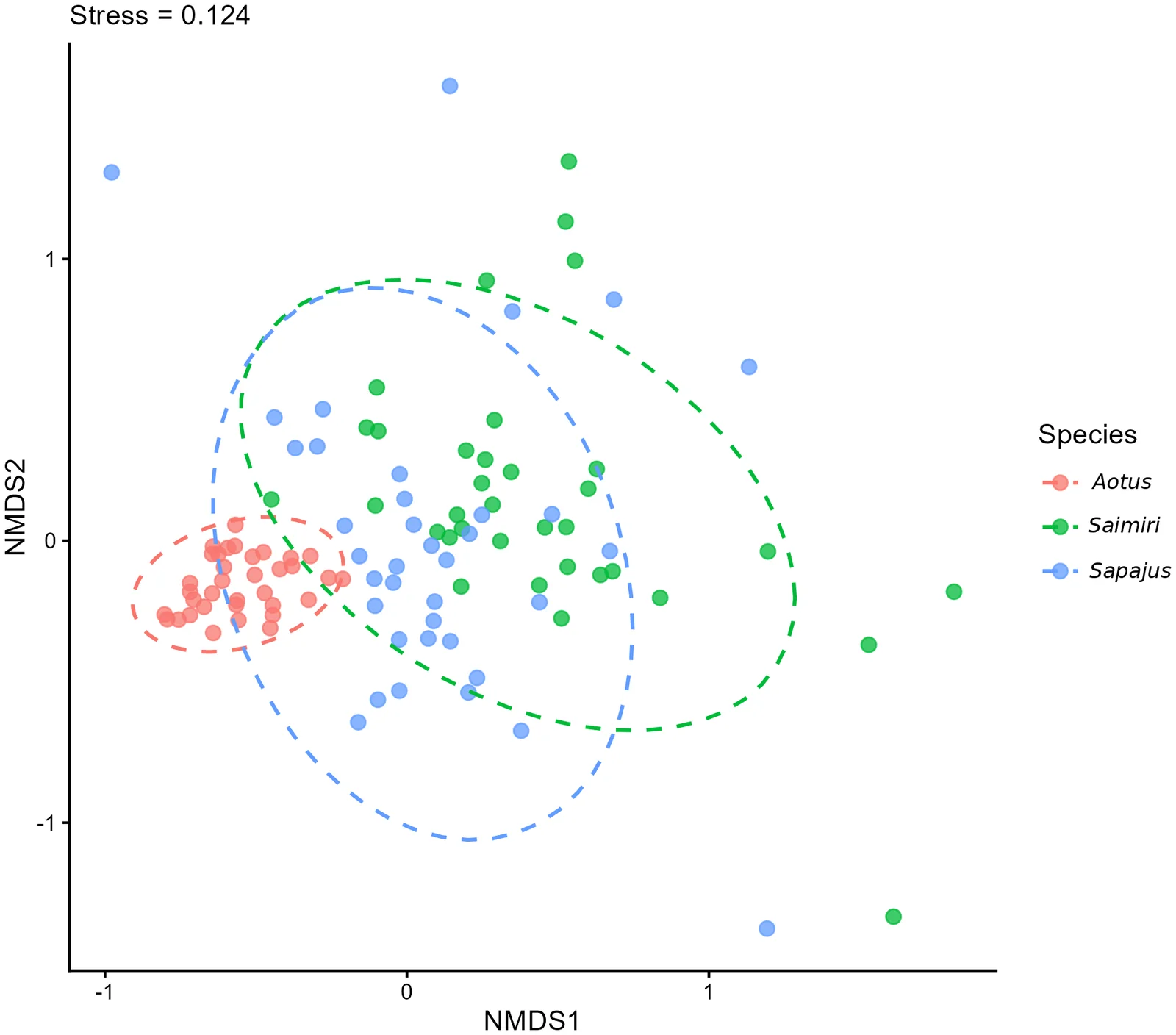
Distances derived from dermatoglyphic and delta analyses of the plantar limbs of *Aotus infulatus*, *Saimiri collinsi*, and *Sapajus* spp. Each point represents one individual. Points with different colors and shapes represent samples from different taxa and antimeres, respectively. Stress indicates the goodness-of-fit of the non-metric multidimensional scaling (nMDS) model. Source: Graph produced using R Core Team software [22].

### 3.2. Level II dermatoglyphic analysis

Regarding Level II analysis (minutiae), a total of 39,258 minutiae were analyzed in the hypothenar region of palmar and plantar surfaces of the studied taxa, including 28,542 minutiae in *A. infulatus*, 2,271 in *S. collinsi*, and 8,445 in *Sapajus* spp.

Seventeen types of minutiae were identified in the samples: ridge ending (Fig 5A), bifurcation (Fig 5B), opposite bifurcation (Fig 5C), big and small fragment (both represented in Fig 5D), break (Fig 5E), dot between ridges P-BW (Fig 5F), dot on the ridge P-IN (Fig 5G), overlap (Fig 5H), crossbar (Fig 5I), bridge (Fig 5J), dock (Fig 5K), trifurcation (Fig 5L), M (Fig 5M), return (Fig 5N), and big and small enclosure (both represented in Fig 5O). Big and small fragments and big and small enclosures were considered separate minutia types, resulting in a total of 17 minutia types.

**Fig 5:**
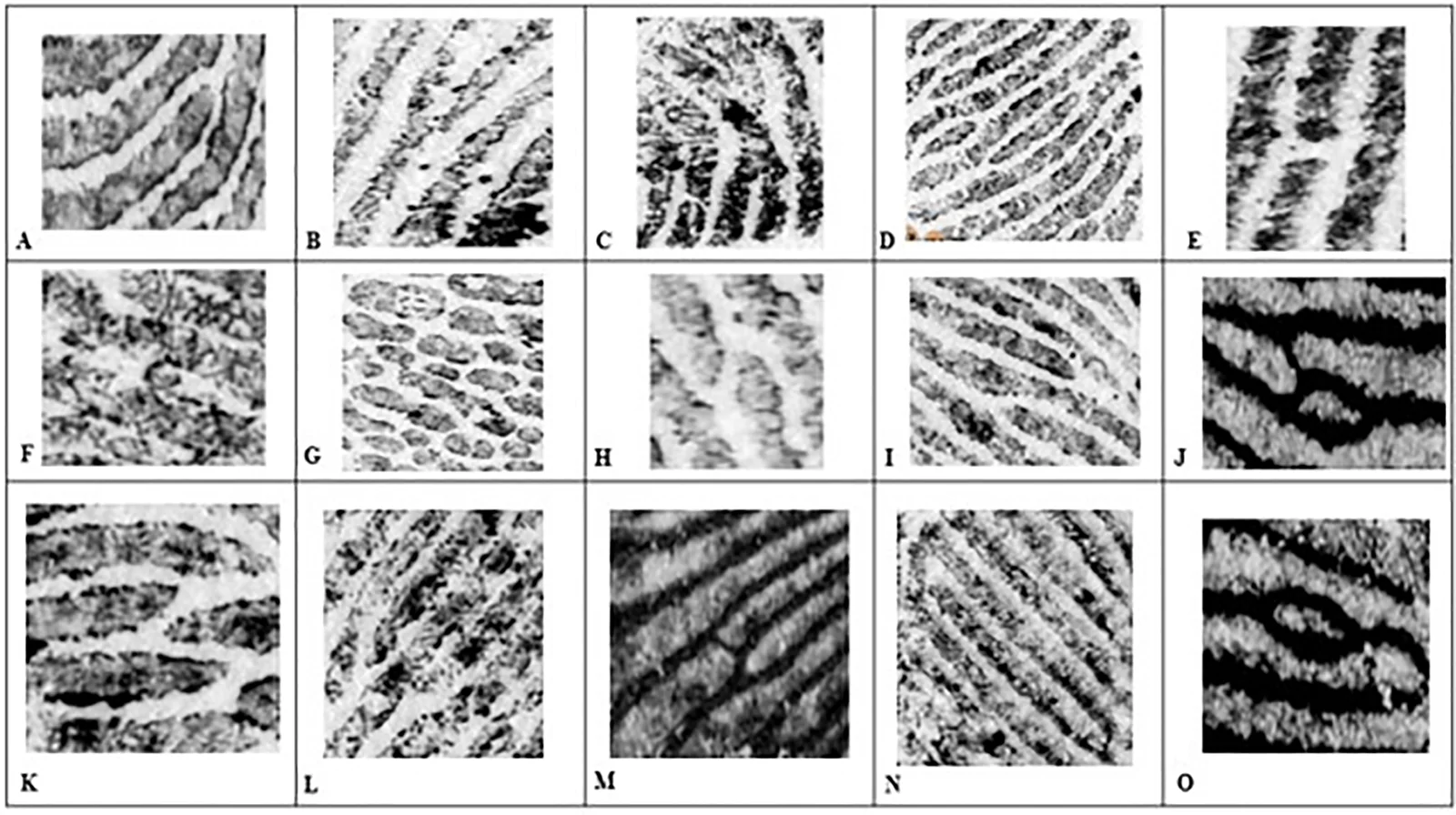
Types of minutiae identified in the palmar and plantar hypothenar regions of *Sapajus* spp., *Saimiri collinsi*, and *Aotus infulatus.* Legend: ridge ending (A), bifurcation (B), opposite bifurcation (C), big and small fragment (D), break (E), dot between ridges P-BW (F), dot on the ridge P-IN (G), overlap (H), crossbar (I), bridge (J), dock (K), trifurcation (L), M (M), return (N), and big and small enclosure (O).

The main types of minutiae observed in the palmar and plantar epidermal impressions of *Sapajus* spp., *S. collinsi*, and *A. infulatus* are presented in Table 6. Bridge, opposite bifurcation, M, and trifurcation minutiae were identified in only a few samples. The bridge minutia was recorded in four thoracic limb (4/54; 7.4%) and one pelvic limb (1/40; 2.5%) impressions of *Sapajus* spp., and in three thoracic limb (3/68; 4.4%) and one pelvic limb (1/66; 1.5%) impressions of *A. infulatus*. Opposite bifurcation occurred in two thoracic limb (2/54; 3.7%) and one pelvic limb (1/40; 2.5%) impressions of *Sapajus* spp., and in two thoracic limb (2/68; 2.9%) and three pelvic limb (3/66; 4.5%) impressions of *A. infulatus*. Finally, the M was recorded in three thoracic limb impressions (3/54; 5.5%) of *Sapajus* spp. and in one thoracic limb impression (1/68; 1.5%) of *A. infulatus*, whereas trifurcation was observed only in one pelvic limb impression (1/66; 1.5%) of *A. infulatus*.

**Table 6:** Level II dermatoglyphic analysis (minutiae) of the palmar and plantar hypothenar regions of *Aotus infulatus*, *Saimiri collinsi*, and *Sapajus* spp.

Mi	T	LTL	RTL	TL	LPL	RPL	PL
B	Sa	100%; 8.52 ± 4.6	100%; 8.74 ± 4.42	100%; 8.63 ± 4.47	100%; 9.35 ± 5.55	100%; 10.2 ± 5.86	100%; 9.78 ± 5.65^*#^
	Sm	93.3%; 3.73 ± 3.15	90%; 4.2 ± 3.76	92%; 3.92 ± 3.34	90.9%; 3.73 ± 3.10	90%; 2.5 ± 1.71	90.5%; 3.14 ± 2.55 ^+ #^
	Ao	82.3%;2.59 ± 2.41	70.1%; 2.26 ± 2.49	76.7%; 2.43 ± 2.44	69.7%; 2.54 ± 2.6	69.7%; 2.03 ± 2.73	69.7%; 2.29 ± 2.66^*+^
O	Sa	51.8%. 0.93 ± 1.11	51.8%. 0.96 ± 1.22	51.8%. 0.94 ± 1.16	70%. 1.25 ± 1.07	70%. 1.25 ± 1.33	70%. 1.25 ± 1.19^#^
	Sm	40%; 0.67 ± 0.99	30%; 0.5 ± 0.97	36%; 0.6 ± 0.96	45.4%; 1 ± 1.34	20%; 0.6 ± 1.35	33.3%; 0.81 ± 1.33^#^
	Ao	67.6%; 1.73 ± 1.91	76.5%; 1.35 ± 1.18	72.1%; 1.54 ± 1.59	63.6%; 1.45 ± 1.5	72.7%;1.61 ± 1.52	68.2%;1.53 ± 1.5
EN-BG	Sa	18.5%; 0.18 ± 0.4	18.5%; 0.26 ± 0.59	18.5%; 0.22 ± 0.5	20%; 0.35 ± 0.93	20%; 0.2 ± 0.41	20%; 0.27 ± 0.72^#^
	Sm	(0)	10%; 0.1 ± 0.32	4%; 0.04 ± 0.2	(0)	(0)	(0) ^#^
	Ao	(0)	5.9%; 0.06 ± 0.24	2.9%; 0.03 ± 0.17	(0)	3%; 0.03 ± 0.17	1.4%; 0.01 ± 0.12
D	Sa	11.1%;0.15 ± 0.46	(0)	5.5%; 0.07 ± 0.33	5%; 0.05 ± 0.22	20%; 0.2 ± 0.41	12.5%; 0.12 ± 0.33^*^
	Sm	(0)	10%; 0.1 ± 0.32	4%; 0.04 ± 0.2	(0)	(0)	(0) ^+^
	Ao	47.05%. 0.97 ± 1.38	47.05%. 0.79 ± 1.20	47.05%. 0.88 ± 1.29	39.4%; 0.58 ± 0.83	36.4%; 0.61 ± 1	37.9%; 0.59 ± 0.91^*+^
EN-SM	Sa	22.2%; 0.3 ± 0.61	37.04%; 0.52 ± 0.75	29.6%; 0.41 ± 0.69	30%; 0.45 ± 0.82	30%; 0.3 ± 0.47	30%; 0.37 ± 0.67^#^
	Sm	20%;0.2 ± 0.4	(0)	12%; 0.12 ± 0.33	(0)	(0)	(0) ^+#^
	Ao	2.9%; 0.03 ± 0.17	5.9%; 0.06 ± 0.24	4.4%; 0.04 ± 0.21	9.1%; 0.12 ± 0.41	9.1%; 0.15 ± 0.57	9.1%; 0.14 ± 0.49^+^
F-SM	Sa	92.3%; 2.78 ± 2.34	85.2%; 2.44 ± 2.01	88.9%; 2.61 ± 2.17	85%; 2.65 ± 3.27	80%; 2.25 ± 1.86	82.5%; 2.45 ± 2.63^#^
	Sm	40%; 0.87 ± 1.46	30%; 0.7 ± 1.25	36%; 0.8 ± 1.35	9.1%; 0.09 ± 0.3	40%; 0.6 ± 0.84	23.8%; 0.33 ± 0.66^#^
	Ao	85.3%; 3.09 ± 2.23	79.4%; 2.41 ± 2.36	82.3%; 2.75 ± 2.31	93.9%; 5.48 ± 3.89	100%; 4.42 ± 3.56	94.1%; 4.95 ± 3.74
BR	Sa	100%;8.7 ± 5.08	96.3%; 7.96 ± 4.85	98.1%; 8.35 ± 4.93	95%; 8.5 ± 8.44	100%; 8.3 ± 4.12	97.5%; 8.4 ± 6.56^#^
	Sm	86.7%;3.87 ± 3.04	80%; 3.6 ± 4.32	84%; 3.76 ± 3.53	81.8%; 4.36 ± 4.76	90%; 4.9 ± 3.03	85.7%; 4.62 ± 3.94 ^+ #^
	Ao	97.05%; 4.44 ± 3.37	88.2%; 2.76 ± 2.28	92.6%; 3.6 ± 2.98	84.8%; 3.78 ± 2.88	84.8%; 3.24 ± 2.67	84.8%; 3.5 ± 2.77^+^
F-BG	Sa	88.9%; 3.22 ± 2.65	92.6%; 4.15 ± 2.43	90.7%; 3.68 ± 2.56	75%; 2.85 ± 3.1	95%; 4.6 ± 3.70	85%; 3.72 ± 3.49^#^
	Sm	40%;0.53 ± 0.74	79%; 0.9 ± 0.74	52%; 0.68 ± 0.75	18.2%; 0.18 ± 0.4	40%; 0.5 ± 0.71	28.6%; 0.33 ± 0.58 ^+ #^
	Ao	97.05%; 5.62 ± 3.85	100%; 4.82 ± 4.29	98.5%; 5.22 ± 4.07	100%; 8.97 ± 5.82	100%; 8.91 ± 6.99	100%; 8.94 ± 6.39^+^
P-IN	Sa	70.4%. 21.3 ± 21.1	85.2%. 21 ± 16.84	77.8%. 21.15 ± 18.91	40%. 19 ± 33.31	45%. 8.35 ± 13.64	42.5%. 13.67 ± 25.69^*#^
	Sm	26.7%; 22.4 ± 14.31	30%; 14.1 ± 13.01	28%; 19.08 ± 14.15	9.1%; 0.45 ± 1.51	10%; 1 ± 3.16	9.5%; 0.71 ± 2.39 ^+ #^
	Ao	100%; 116.15 ± 71.16	97.1%; 84.44 ± 57.07	98.5%; 100.29 ± 65.98	100%; 151.51 ± 95.1	100%; 147.51 ± 93.57	100%; 149.51 ± 94.08^*+^
P-BW	Sa	51.8%. 0.89 ± 1.19	59.2%. 0.89 ± 0.97	55.5%. 0.89 ± 1.08	30%. 0.8 ± 1.64	45%. 1.2 ± 1.64	37.5%. 1 ± 1.63^#^
	Sm	26.7%; 0.27 ± 0.46	30%; 0.3 ± 0.48	28%; 0.28 ± 0.46	9.1%; 0.09 ± 0.30	10%; 0.1 ± 0.32	9.5%; 0.09 ± 0.3^#^
	Ao	35.3%; 1.03 ± 1.83	35.3%; 0.94 ± 1.92	35.3%; 0.98 ± 1.86	48.5%; 1.09 ± 1.81	42.4%; 1 ± 1.5	45.4%; 1.04 ± 1.65
E	Sa	100%; 44 ± 19.11	100%; 49.89 ± 20.3	100%; 46.94 ± 19.75	100%; 41.05 ± 14.29	100%; 44.15 ± 27.24	100%; 42.6 ± 21.53^#^
	Sm	100%; 25.93 ± 9.58	100%; 28.4 ± 8.92	100%; 26.92 ± 9.21	100%; 31.36 ± 9.78	100%; 30.7 ± 8.79	100%; 31.05 ± 9.1 ^+ #^
	Ao	100%; 59.91 ± 18.16	100%; 56.03 ± 15.49	100%; 57.97 ± 16.87	100%; 77.15 ± 23.31	100%; 76.67 ± 20.55	100%; 76.91 ± 21.81^+^
CR	Sa	(0)	3.7%. 0.04 ± 0.19	1.8%. 0.02 ± 0.14	(0)	(0)	(0)
	Sm	(0)	(0)	(0)	(0)	(0)	(0)
	Ao	2.9%; 0.03 ± 0.17	2.9%; 0.03 ± 0.17	2.9%; 0.03 ± 0.17	6.1%; 0.06 ± 0.24	12.1%; 0.12 ± 0.33	9.1%; 0.09 ± 0.29
R	Sa	18.5%.0.18 ± 0.4	14.8%. 0.18 ± 0.48	16.7%. 0.18 ± 0.44	10%. 0.1 ± 0.31	10%. 0.1 ± 0.31	10%. 0.1 ± 0.30^*#^
	Sm	6.7%; 0.07 ± 0.26	(0)	4%; 0.04 ± 0.2	9.1%;0.09 ± 0.30	(0)	4.8%; 0.05 ± 0.22^#^
	Ao	47.1%; 0.82 ± 1.19	41.2%; 0.88 ± 1.47	44.1%; 0.85 ± 1.33	39.4%; 0.61 ± 0.93	39.4%; 0.85 ± 1.35	39.4%; 0.73 ± 1.16^*^

Legend: *Indicates a statistically significant difference (p < 0.05) between *A. infulatus* and *Sapajus* spp.; ^+^Indicates a statistically significant difference (p < 0.05) between *A. infulatus* and *S. collinsi*; ^#^Indicates a statistically significant difference (p < 0.05) between *Sapajus* spp. and *S. collinsi*; Ao: *A. infulatus* (68 TL and 66 PL); B: bifurcation; BR: break; CR: crossbar; D: dock; E: ridge ending; EN-BG: big enclosure; EN-SM: small enclosure; F-BG: big fragment; F-SM: small fragment; LPL: left pelvic limb; LTL: left thoracic limb; Mi: minutiae; O: overlap; P-BW: dot between ridges; P-IN: dot on the ridge; PL: pelvic limbs (right and left); R: return; RPL: right pelvic limb; RTL: right thoracic limb; Sa: *Sapajus* spp. (54 TL and 40 PL); Sm: *Saimiri collinsi* (25 TL and 21 PL); T: taxon; TL: thoracic limbs (right and left). Data are presented as: frequency of animals exhibiting the minutiae type/total number of samples (%); mean ± standard deviation of the number of each minutiae type.

Regarding minutiae frequency, ridge ending was the most frequent minutia, with 100% occurrence in all analyzed taxa and limbs. Bifurcation also showed high representativeness in *Sapajus* spp. and *S. collinsi*, with overall frequencies of 100% and 91.3%, respectively. In contrast, dot on the ridge P-IN was the most prominent minutia in *A. infulatus*, with a frequency of 99.2%.

When investigating differences in minutiae patterns, statistically significant effects were observed for taxon (F = 37.550; p = 0.001), and region (palmar or plantar) (F = 2.506; p = 0.0017). However, the coefficient of determination associated with region was very low (R² = 0.0189), indicating reduced explanatory effects for these variables. No clear difference was detected between antimers (R^2^ = 0.0049, F = 1.291, p = 0.0519), and the interaction between anatomical region and side was not significant (R^2^ = 0.0050, p = 0.4856). Likewise, sex (R^2^ = 0.0073, F = 1.132, p = 0.2846), age category (R^2^ = 0.0045, F = 0.702, p = 0.4538), and their interaction (R^2^ = 0.0042, F = 0.647, p = 0.4900) were not statistically significant. Therefore, only taxon identity (R^2^ = 0.4827) was considered a substantial effect with potential biological relevance.

The variable that most strongly contributed to discriminating *A. infulatus* from *Sapajus* spp. and *S. collinsi* was dot on the ridge P-IN, with mean dissimilarities of 0.33 ± 0.17 (p = 0.001) and 0.39 ± 0.17 (p = 0.001), respectively. In the comparison between *Sapajus* spp. and *S. collinsi*, the most relevant variable was ridge ending, with a mean dissimilarity of 0.14 ± 0.10 (p = 0.001) (Table 7, S3 Table in S1 File).

**Table 7:** Variables contributing to the dissimilarity among *Aotus infulatus*, *Saimiri collinsi*, and *Sapajus* spp. in the Level II palmar and plantar dermatoglyphic analysis.

Taxa	Minutiae	Mean	SD	Ratio	p*
*Aotus × Sapajus*	Dot on the ridge (P-IN)	0.3236	0.1508	2.15	<0.001
	Return	0.0023	0.0019	1.25	<0.001
	Dock	0.0023	0.0020	1.12	<0.001
*Aotus × Saimiri*	Dot on the ridge (P-IN)	0.3981	0.1470	2.71	<0.001
	Ridge ending	0.1533	0.0539	2.84	<0.001
	Big fragment	0.0237	0.0109	2.17	<0.001
	Small fragment	0.0134	0.0084	1.61	<0.001
	Return	0.0030	0.0022	1.34	<0.001
	Dock	0.0028	0.0024	1.15	<0.001
*Saimiri × Sapajus*	Ridge ending	0.1312	0.0894	1.47	<0.001
	Bifurcation	0.0404	0.0263	1.53	<0.001
	Break	0.0368	0.0260	1.42	<0.001
	Big fragment	0.0216	0.0116	1.86	<0.001
	Small fragment	0.0160	0.0097	1.65	<0.001
	Overlap	0.0062	0.0045	1.37	<0.001
	Dot between ridges (P-BW)	0.0060	0.0047	1.28	<0.001
	Small enclosure	0.0030	0.0026	1.14	<0.001
	Big enclosure	0.0017	0.0024	0.73	<0.001

Legend: *p-values were obtained from permutation tests associated with the SIMPER analysis. Mean represents the average contribution of each minutia type to the dissimilarity between groups; SD is the standard deviation of this contribution; Ratio = Mean/SD. P-values were obtained Only minutiae with p < 0.05 are presented.

Similarly to the pattern observed in the Level I dermatoglyphic analysis, high intrataxon variation was detected (F = 14.714; p = 0.0001). In addition, minutiae patterns also varied among species, with mean distances to centroid of 0.153 for *A. infulatus*, 0.316 for *S. collinsi*, and 0.221 for *Sapajus* spp. (Fig 6).

**Fig 6:**
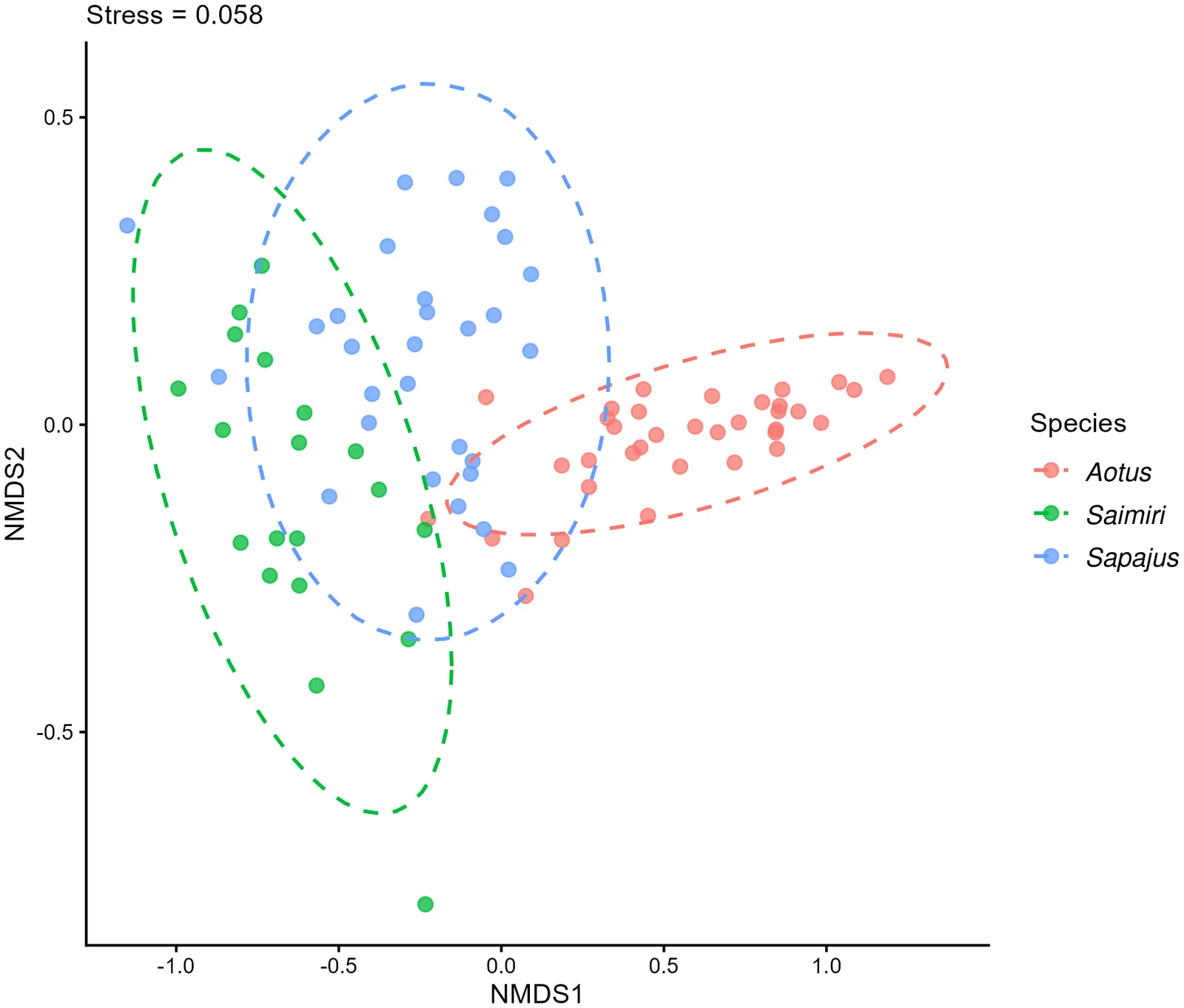
Distances derived from minutiae analyses of the palmar and plantar hypothenar regions of *Aotus infulatus*, *Saimiri collinsi*, and *Sapajus* spp. Points with different colors and shapes represent samples from different taxa and regions, respectively. Stress indicates the goodness-of-fit of the non-metric multidimensional scaling (nMDS) model. Source: Graph produced using R Core Team software [22].

In the Level II dermatoglyphic analysis of the hypothenar region, no identical minutiae configurations were observed among individuals within the analyzed sample. Although some animals showed similar configurations, all pairwise comparisons presented at least one divergent variable. The analysis of 147 palmar and 127 plantar epidermal impressions from A. infulatus, S. collinsi, and Sapajus spp. confirmed the presence of at least 12 minutiae in all analyzed regions. These findings indicate substantial individual-level variability in palmar and plantar friction ridge patterns and support their further investigation in future validation studies.

## 4. Discussion

The lower intrataxon variation in dermatoglyphic patterns observed in *A. infulatus* compared with *S. collinsi* and *Sapajus* spp. may be related to differences in activity pattern, locomotor behavior, substrate use, and manipulative ability. *S. collinsi* and *Sapajus* spp. are diurnal non-human primates characterized by complex fine manipulation and agile exploratory behavior, which may expose their hands and feet to more variable mechanical loading and potentially contribute to greater heterogeneity in delta and dermatoglyphic patterns [4,8,23–25]. In contrast, *A. infulatus* is a nocturnal and predominantly arboreal species with a relatively conservative locomotor and manipulative repertoire, including movements along stable and repeatedly used routes [2,26]. These ecological and behavioral differences provide hypotheses for future comparative studies. However, because these characteristics were confounded with taxon identity, the present design does not allow their independent contributions to be determined. Therefore, the observed differences should be interpreted as taxon-related associations rather than evidence of ecological or functional causation.

A study involving *Callicebus* sp. reported dermatoglyphic patterns characterized by the predominance of whorls on palmar surfaces, especially in the interdigital region, and loops on plantar surfaces [27]. In *Cacajao* sp., loops were described as the predominant dermatoglyphic pattern in both hands and feet, whereas whorls were restricted to some palmar interdigital areas [28]. Similarly, in *Macaca fascicularis*, a higher frequency of whorls was observed in palmar regions (37.9%) [29]. In the present study, *Sapajus* spp. and *S. collinsi* exhibited a higher frequency of whorls on the hands (60.5% and 80%, respectively) and loops on the feet (41% and 46.4%, respectively), partially consistent with findings reported for other non-human primate species. In contrast, *A. infulatus* showed predominance of whorls in all limbs (99.5% TL and 96.7% PL), suggesting a more uniform dermatoglyphic organization possibly associated with specific functional demands.

The higher mean number of deltas observed in the hands of *Sapajus* spp. compared with *S. collinsi* may be associated with differences in the organization of the friction ridge patterns and, consequently, in the configuration of the palmar contact surface. Although such differences may be functionally relevant to hand–substrate interactions, the number of deltas alone cannot be interpreted as a direct indicator of manual dexterity. Manual dexterity is a complex, multifactorial trait involving not only cutaneous and frictional properties of the hand but also the morphology and functional integration of bones, muscles, and joints, as well as neural mechanisms underlying motor control and sensory innervation. Nevertheless, the behavioral differences between these taxa provide relevant context for interpreting the observed dermatoglyphic variation. *Sapajus* spp. routinely uses multiple forms of precision grip through functional opposition between the thumb and index finger, whereas *S. collinsi* predominantly performs palmar grasps involving the entire hand, with hand use more closely associated with agile locomotion and rapid food capture [6,23–25,30,31].Seventeen types of minutiae were identified at different frequencies among the taxa evaluated in the present study. Similarly, in a chiroscopic and podoscopic analysis performed in female *Chlorocebus aethiops*, Tobelem [32] recorded 14 types of minutiae. In turn, Herter et al. [14] employed a model adapted from Gutiérrez et al. [33] and classified only six categories of minutiae in palmar and plantar samples of *C. penicillata*. Conversely, Gutiérrez-Redomero et al. [34] performed a detailed analysis of the frequency and distribution of digital minutiae in a Spanish human population and proposed the presence of 20 different types. In that study, consideration of minutiae orientation (clockwise or counterclockwise) increased the number of observed categories, since it distinguished, for example, clockwise bifurcations from counterclockwise convergences [34].

In studies by Gutiérrez et al. [33] and Gutiérrez-Redomero et al. [34] involving human fingerprints, and by Kondeková et al. [35] involving human palmar impressions, the most frequently recorded minutia was ridge ending, with frequencies ranging from 40% to 60%. Additionally, Gutiérrez-Redomero et al. [34] reported that bifurcations and convergences, frequently analyzed together in most studies, showed frequencies between 18% and 40%. In palmar and plantar impressions of *C. penicillata*, Herter et al. [14] observed a similar predominance of ridge ending, with frequencies exceeding 80%. The authors also highlighted the frequent occurrence of fragment and bifurcation minutiae. Corroborating these findings, the present study identified ridge ending as the most frequent minutia in all analyzed taxa and limbs. Bifurcation also showed high representativeness in *Sapajus* spp. and *S. collinsi*, with overall frequencies of 100% and 91.3%, respectively, whereas in *A. infulatus*, dot on the ridge P-IN was the most prominent minutia, with a frequency of 99.2%. The predominance of P-IN in *A. infulatus* represents a taxon-associated pattern that warrants further investigation. Although *Aotus* genus is distinguished from most other Neotropical primates by its nocturnal activity, the present design does not allow this dermatoglyphic pattern to be attributed independently to nocturnality or to any other ecological characteristic. This reinforces the need for expanded sampling within the genus to support more robust ecological investigations.

Consistent with the findings of Tobelem [32] in dactyloscopic analyses of female *C. aethiops*, and Herter et al. [14] in palmar and plantar impressions of male and female *C. penicillata*, the present study found no identical patterns among palmar and plantar impressions of different individuals across the three evaluated taxa. These results are consistent with previous observations that palmar and plantar friction ridge patterns may show individual-level variability in non-human primates [14,32], although formal validation studies are still required before their use as an individual identification method can be established, as has been more extensively documented in human friction ridge analysis [36].

In agreement with Herter et al. [14], PERMANOVA did not detect a significant effect of sex on minutiae composition (R^2^ = 0.0073, p = 0.2846). In contrast, several studies in human populations have reported sexual dimorphism both in the total number and in the frequency of specific minutiae types, depending on the population and anatomical region analyzed [33,34,36,37]. Dermatoglyphic sexual dimorphism has been associated with sex-linked genetic and ancestry-related factors expressed during embryonic development of friction ridges, rather than postnatal influences such as hand size or environmental conditions. In the present study, the smaller sample size, the use of palmar and plantar regions instead of digits, and the restriction of analyses to the hypothenar region may have reduced statistical power, contributing to the absence of sexual dimorphism in the three analyzed taxa. Furthermore, although non-human primates exhibit social organization in groups, activities and environmental exposure are broadly shared between males and females, which may be insufficient to generate selective pressures capable of producing distinct dermatoglyphic patterns between sexes.

This study provides novel data on intra- and intertaxon variation in Level I and Level II dermatoglyphic characteristics of the palmar and plantar surfaces of non-human primates. The differences observed among taxa, particularly between *A. infulatus* and the other analyzed groups, demonstrate taxon-related variation in dermatoglyphic expression. Although these taxa also differ in several ecological and behavioral characteristics, the present design does not allow the effects of these factors to be separated from taxon identity. Studies including a broader range of taxa with overlapping ecological characteristics are required to investigate potential ecological, functional, genetic, and environmental associations.

The present findings may also provide a foundation for future computational approaches to non-human primate dermatoglyphic analysis. In humans, recent advances have shown that artificial intelligence can be applied to large-scale fingerprint datasets for pattern classification, pose estimation, minutiae detection, and statistical modeling of minutiae frequency and spatial distribution [38]. Although the present study was not designed to validate automated identification or taxonomic classification, the observed taxon-related variation in Level I and Level II traits suggests that palmar and plantar friction ridge impressions may represent structured biometric data for future machine learning applications. Larger reference databases combining high-resolution images, manually validated minutiae maps, anatomical region labels, and taxonomic metadata could support models aimed at taxon classification, individual re-identification, and image-quality screening. Such approaches may be useful in primate research centers, conservation programs, wildlife forensic investigations, and cases involving seized animals or carcasses in which palmar or plantar skin is preserved.

## 5. Study limitations

In the present study, the inclusion of individuals from only three taxa limited broader ecological inferences. Therefore, increasing both taxonomic representation and the number of individuals per group in future investigations would strengthen ecological comparisons. Another limitation was the exclusion of some samples due to compromised image quality. The implementation of more rigorous methodological protocols, together with appropriate training of collaborators, may help minimize such limitations, particularly in Level II analyses.

Moreover, ecological interpretations should be regarded as exploratory rather than causal. In the present design, taxon identity was confounded with locomotor behavior, diet, activity pattern, forest stratum use, and manipulative ability, preventing the independent contribution of each ecological factor from being disentangled. In addition, because the analyzed individuals were maintained under human care, environmental conditions, substrate use, and management practices may have influenced the quality or expression of palmar and plantar friction ridge impressions. Finally, the absence of coincident minutiae configurations within the analyzed sample should not be interpreted as a formal validation of individual identification. Future studies should include broader taxonomic sampling, standardized acquisition protocols, blind validation procedures, and estimates of interobserver agreement, intraobserver repeatability, and error rates under different collection conditions.

## 6. Conclusion

Level I and Level II dermatoglyphic analyses of the palmar and plantar regions of *A. infulatus*, *S. collinsi*, and *Sapajus* spp. revealed consistent taxon-related variation, particularly between *A. infulatus* and the other analyzed groups. *A. infulatus* showed a more homogeneous dermatoglyphic profile, with higher frequencies of deltas and whorls, whereas ridge endings were the most frequent Level II minutiae across all taxa and limbs (S1 Tables in S1 File and S2 Files). In addition, no coincident palmar or plantar epidermal impressions were observed among individuals within the analyzed sample, indicating substantial individual-level variability in these structures. These findings support the use of palmar and plantar friction ridge patterns as morphological markers for comparative studies in non-human primates and suggest their potential relevance for future biometric, management, and conservation applications, provided that their discriminatory performance is formally validated.

## Supporting information

S1 File Appendix. PERMANOVA results for Level I analysis of palmar and plantar epidermal impressions of *Aotus infulatus*, *Saimiri collinsi*, and *Sapajus* spp. andresults for minutiae of palmar and plantar epidermal impressions in the hypothenar region of *Aotus infulatus*, *Saimiri collinsi*, and *Sapajus* spp.
(DOCX)

S2 File Data used for statistical analyses. This dataset contains individual-level data on palmar and plantar friction ridge patterns in *Aotus infulatus*, *Saimiri collinsi*, and *Sapajus* spp.
(XLSX)
