# Supplementary material for: Taxon-related variation in palmar and plantar friction ridge patterns among Aotus infulatus, Saimiri collinsi and Sapajus spp.: Individual-level variation and implications for identification
Source: PLoS One. 2026 Nov 11;21(11):e0360621. doi: 10.1371/journal.pone.0360621 (PMC13754117; doi:10.1371/journal.pone.0360621)
Supplement: Supplementary file 1 [file pone.0360621.s001.docx]

APPENDIX I – Level I papiloscopic analyses

Table 1. PERMANOVA results for Level I analysis of palmar epidermal impressions of *Aotus infulatus*, *Saimiri collinsi*, and *Sapajus* spp.

| **Taxa** | **Region/ Level I analysis** | **Mean** | **SD** | **Ratio** | **p*** |
| --- | --- | --- | --- | --- | --- |
| *Aotus x Sapajus* | Hypothenar/delta | 0.08958 | 0.04910 | 182.450 | 0.001 |
|  | Hypothenar/whorl | 0.07063 | 0.02388 | 295.750 | 0.001 |
|  | Interdigital/delta | 0.04871 | 0.04271 | 114.030 | 1.000 |
|  | Thenar/delta | 0.03289 | 0.02625 | 125.290 | 0.932 |
|  | Thenar/whorl | 0.01753 | 0.01881 | 0.93200 | 0.001 |
|  | Hypothenar/loop | 0.01649 | 0.01745 | 0.94510 | 0.347 |
|  | Thenar/loop | 0.01366 | 0.01556 | 0.87790 | 0.001 |
|  | Interdigital/whorl | 0.00813 | 0.01812 | 0.44880 | 0.993 |
|  | Interdigital/arch | 0.00477 | 0.01118 | 0.42660 | 0.001 |
|  | Thenar/arch | 0.00218 | 0.00976 | 0.22330 | 0.306 |
|  | Hypothenar/arch | 0.00161 | 0.00678 | 0.23790 | 0.698 |
|  | Interdigital/loop | 0.00040 | 0.00336 | 0.12000 | 0.001 |
| *Aotus x Saimiri* | Hypothenar/delta | 0.10882 | 0.05271 | 206.450 | 0.001 |
|  | Interdigital/delta | 0.08193 | 0.06088 | 134.580 | 0.001 |
|  | Hypothenar/whorl | 0.06898 | 0.03578 | 192.810 | 0.001 |
|  | Thenar/delta | 0.03790 | 0.02906 | 130.430 | 0.023 |
|  | Thenar/loop | 0.01335 | 0.01606 | 0.83120 | 0.002 |
|  | Thenar/whorl | 0.01172 | 0.01726 | 0.67900 | 0.985 |
|  | Interdigital/whorl | 0.01054 | 0.02465 | 0.42750 | 0.832 |
|  | Hypothenar/loop | 0.00589 | 0.01237 | 0.47640 | 1.000 |
|  | Interdigital/arch | 0.00421 | 0.01111 | 0.37930 | 0.079 |
|  | Hypothenar/arch | 0.00224 | 0.00826 | 0.27090 | 0.303 |
|  | Thenar/arch | 0.00088 | 0.00516 | 0.17060 | 0.845 |
|  | Interdigital/loop | 0.00043 | 0.00356 | 0.11980 | 0.001 |
| *Sapajus x Saimiri* | Interdigital/delta | 0.09198 | 0.07819 | 117.630 | 0.001 |
|  | Hipotenar/delta | 0.04967 | 0.04444 | 111.760 | 1.000 |
|  | Thenar/delta | 0.03785 | 0.03630 | 104.260 | 0.010 |
|  | Hypothenar/whorl | 0.03443 | 0.03413 | 100.870 | 1.000 |
|  | Hypothenar/loop | 0.02500 | 0.02689 | 0.92990 | 0.001 |
|  | Interdigital/whorl | 0.01825 | 0.03965 | 0.46040 | 0.001 |
|  | Thenar/whorl | 0.01551 | 0.02265 | 0.68490 | 0.143 |
|  | Thenar/loop | 0.00784 | 0.01807 | 0.43370 | 1.000 |
|  | Thenar/arch | 0.00195 | 0.01236 | 0.15790 | 0.409 |
|  | Hypothenar/arch | 0.00177 | 0.00869 | 0.20370 | 0.581 |
|  | Interdigital/arch | 0.00157 | 0.00808 | 0.19460 | 0.997 |
|  | Interdigital/loop | 0.00000 | 0.00000 | NA | NA |

Legend: *p-values were obtained by PERMANOVA using a significance level of 5%. “Mean” represents the average contribution of each variable to the dissimilarity among groups, whereas SD indicates the standard deviation of this contribution, both obtained from the SIMPER analysis. “Ratio” corresponds to the relationship between the mean contribution and its standard deviation (Mean/SD), indicating the consistency of each variable in discriminating among taxa. NA: not applicable due to absence of ocurrences.

Table 2. PERMANOVA results for Level I analysis of plantar epidermal impressions of *Aotus infulatus*, *Saimiri collinsi*, and *Sapajus* spp.

| **Taxa** | **Region/ Level I analysis** | **Mean** | **SD** | **Ration** | **p*** |
| --- | --- | --- | --- | --- | --- |
| *Aotus x Sapajus* | Hypothenar/delta | 0.10155 | 0.06034 | 168.280 | 0.001 |
|  | Hypothenar/whorl | 0.06546 | 0.03287 | 199.150 | 0.001 |
|  | Interdigital/delta | 0.05585 | 0.04189 | 133.330 | 1.000 |
|  | Thenar/delta | 0.05286 | 0.04426 | 119.440 | 1.000 |
|  | Interdigital/whorl | 0.03951 | 0.02930 | 134.860 | 0.925 |
|  | Thenar/whorl | 0.03595 | 0.02385 | 150.720 | 0.001 |
|  | Thenar/loop | 0.03002 | 0.02270 | 132.230 | 1.000 |
|  | Hypothenar/loop | 0.02735 | 0.02316 | 118.100 | 0.998 |
|  | Interdigital/loop | 0.01800 | 0.01683 | 106.960 | 1.000 |
|  | Hypothenar/arch | 0.00373 | 0.01082 | 0.34490 | 0.383 |
|  | Interdigital/arch | 0.00208 | 0.00691 | 0.30080 | 0.360 |
|  | Thenar/arch | 0.00204 | 0.00679 | 0.30100 | 0.346 |
| *Aotus x Saimiri* | Hypothenar/delta | 0.12638 | 0.06228 | 202.910 | 0.001 |
|  | Interdigital/delta | 0.08522 | 0.04742 | 179.720 | 0.001 |
|  | Thenar/delta | 0.08187 | 0.05035 | 162.620 | 0.001 |
|  | Hypothenar/whorl | 0.06539 | 0.03915 | 167.040 | 0.001 |
|  | Interdigital/whorl | 0.05124 | 0.03104 | 165.090 | 0.001 |
|  | Thenar/whorl | 0.03884 | 0.02648 | 146.650 | 0.001 |
|  | Hypothenar/loop | 0.03114 | 0.02573 | 121.030 | 0.641 |
|  | Thenar/loop | 0.02538 | 0.02231 | 113.780 | 1.000 |
|  | Interdigital/loop | 0.01816 | 0.01639 | 110.760 | 1.000 |
|  | Interdigital/arch | 0.00000 | 0.00000 | NA | NA |
|  | Thenar/arch | 0.00000 | 0.00000 | NA | NA |
|  | Hypothenar/arch | 0.00000 | 0.00000 | NA | NA |
| *Sapajus x Saimiri* | Thenar/delta | 0.08256 | 0.06612 | 124.870 | 0.001 |
|  | Interdigital/delta | 0.07512 | 0.06487 | 115.790 | 0.001 |
|  | Hypothenar/delta | 0.06826 | 0.05497 | 124.190 | 1.000 |
|  | Thenar/loop | 0.05566 | 0.04657 | 119.530 | 0.001 |
|  | Interdigital/whorl | 0.04610 | 0.04382 | 105.200 | 0.001 |
|  | Interdigital/loop | 0.04018 | 0.03912 | 102.710 | 0.001 |
|  | Hypothenar/loop | 0.03996 | 0.03719 | 107.450 | 0.001 |
|  | Hypothenar/whorl | 0.02603 | 0.03540 | 0.7540 | 1.000 |
|  | Thenar/whorl | 0.02572 | 0.03707 | 0.9380 | 0.999 |
|  | Hypothenar/arch | 0.00648 | 0.01872 | 0.34610 | 0.001 |
|  | Interdigital/arch | 0.00361 | 0.01218 | 0.29610 | 0.001 |
|  | Thenar/arch | 0.00349 | 0.01178 | 0.29670 | 0.001 |

Legend: *p-values were obtained by PERMANOVA using a significance level of 5%. “Mean” represents the average contribution of each variable to the dissimilarity among groups, whereas SD indicates the standard deviation of this contribution, both obtained from the SIMPER analysis. “Ratio” corresponds to the relationship between the mean contribution and its standard deviation (Mean/SD), indicating the consistency of each variable in discriminating among taxa. NA: not applicable due to absence of ocurrences.

APPENDIX II – Level II papiloscopic analyses

Table 3. PERMANOVA results for minutiae of palmar and plantar epidermal impressions in the hypothenar region of *Aotus infulatus*, *Saimiri collinsi*, and *Sapajus* spp.

| **Taxa** | **Minutiae** | **Mean** | **SD** | **Ration** | **p*** |
| --- | --- | --- | --- | --- | --- |
| *Aotus x Sapajus* | Point or dot P-IN | 0.32720 | 0.16918 | 193.420 | 0.001 |
|  | Ridge ending (E) | 0.10500 | 0.07368 | 142.470 | 0.983 |
|  | Bifurcation (B) | 0.02540 | 0.01959 | 129.540 | 0.002 |
|  | Break (BR) | 0.02170 | 0.02034 | 106.550 | 0.787 |
|  | Big fragment (F-BG) | 0.01650 | 0.01371 | 120.440 | 0.998 |
|  | Small fragment (F-SM) | 0.01080 | 0.00997 | 108.210 | 1.000 |
|  | Overlap (O) | 0.00520 | 0.00544 | 0.96390 | 1.000 |
|  | Point or dot P-BW | 0.00520 | 0.00717 | 0.71890 | 0.510 |
|  | Return (R) | 0.00280 | 0.00422 | 0.66720 | 0.008 |
|  | Dock (D) | 0.00260 | 0.00422 | 0.62810 | 0.001 |
|  | Small enclosure (EN-SM) | 0.00160 | 0.00264 | 0.60310 | 0.393 |
|  | Big enclosure (EN-BG) | 0.00090 | 0.00238 | 0.39630 | 0.352 |
|  | Crossbar (CR) | 0.00030 | 0.00101 | 0.25530 | 0.165 |
| *Aotus x Saimiri* | Point or dot P-IN | 0.38950 | 0.16921 | 230.180 | 0.001 |
|  | Ridge ending (E) | 0.15100 | 0.07328 | 206.040 | 0.001 |
|  | Big fragment (F-BG) | 0.02350 | 0.01579 | 148.630 | 0.001 |
|  | Break (BR) | 0.01600 | 0.01580 | 101.250 | 1.000 |
|  | Small fragment (F-SM) | 0.01440 | 0.01268 | 113.710 | 0.001 |
|  | Bifurcation (B) | 0.01370 | 0.01409 | 0.96960 | 1.000 |
|  | Overlap (O) | 0.00650 | 0.00688 | 0.94090 | 0.103 |
|  | Point or dot P-BW | 0.00460 | 0.00849 | 0.53730 | 0.859 |
|  | Return (R) | 0.00310 | 0.00496 | 0.63450 | 0.014 |
|  | Dock (D) | 0.00300 | 0.00503 | 0.59780 | 0.006 |
|  | Small enclosure (EN-SM) | 0.00060 | 0.00180 | 0.34030 | 1.000 |
|  | Crossbar (CR) | 0.00030 | 0.00114 | 0.23500 | 0.249 |
|  | Big enclosure (EN-BG) | 0.00020 | 0.00113 | 0.19730 | 1.000 |
|  | Ridge ending (E) | 0.14479 | 0.09882 | 146.510 | 0.001 |
|  | Point or dot P-IN | 0.12821 | 0.11384 | 112.620 | 1.000 |
|  | Bifurcation (B) | 0.04750 | 0.03659 | 129.820 | 0.001 |
| *Sapajus x Saimiri* | Break (BR) | 0.04405 | 0.03626 | 121.480 | 0.001 |
|  | Big fragment (F-BG) | 0.02319 | 0.01815 | 127.790 | 0.001 |
|  | Small fragment (F-SM) | 0.01672 | 0.01426 | 117.240 | 0.001 |
|  | Overlap (O) | 0.00890 | 0.00862 | 103.320 | 0.001 |
|  | Point or dot P-BW | 0.00668 | 0.00879 | 0.75950 | 0.008 |
|  | Small enclosure (EN-SM) | 0.00315 | 0.00500 | 0.62890 | 0.001 |
|  | Big enclosure (EN-BG) | 0.00183 | 0.00456 | 0.40120 | 0.001 |
|  | Return (R) | 0.00140 | 0.00336 | 0.41640 | 0.999 |
|  | Dock (D) | 0.00077 | 0.00237 | 0.32420 | 1.000 |
|  | Crossbar (CR) | 0.00007 | 0.00065 | 0.10320 | 0.978 |

Legend: *p-values were obtained by PERMANOVA using a significance level of 5%. “Mean” represents the average contribution of each variable to the dissimilarity among groups, whereas SD indicates the standard deviation of this contribution, both obtained from the SIMPER analysis. “Ratio” corresponds to the relationship between the mean contribution and its standard deviation (Mean/SD), indicating the consistency of each variable in discriminating among taxa.
